# Transparent Flexible IGZO Thin Film Transistors Fabricated at Room Temperature

**DOI:** 10.3390/membranes12010029

**Published:** 2021-12-27

**Authors:** Honglong Ning, Xuan Zeng, Hongke Zhang, Xu Zhang, Rihui Yao, Xianzhe Liu, Dongxiang Luo, Zhuohui Xu, Qiannan Ye, Junbiao Peng

**Affiliations:** 1 State Key Laboratory of Luminescent Materials and Devices, Institute of Polymer Optoelectronic Materials and Devices, South China University of Technology, Guangzhou 510640, China; ninghl@scut.edu.cn (H.N.); zengxuanbbj@foxmail.com (X.Z.); zhanghongkekeke@163.com (H.Z.); 201921020680@mail.scut.edu.cn (X.Z.); msyeqiannan@mail.scut.edu.cn (Q.Y.); psjbpeng@scut.edu.cn (J.P.); 2 Research Center of Flexible Sensing Materials and Devices, School of Applied Physics and Materials, Wuyi University, Jiangmen 529020, China; msliuxianzhe@mail.scut.edu.cn; 3 Laboratory for Clean Energy and Materials, Guangzhou Key Huangpu Hydrogen Innovation Center, Institute of Clean Energy and Materials, School of Chemistry and Chemical Engineering, Guangzhou University, Guangzhou 510006, China; luodx2012@163.com; 4 School of Materials and Energy, Guangdong University of Technology, Guangzhou 510006, China; 5 Guangxi Key Lab of Agricultural Resources Chemistry and Biotechnology, Yulin Normal University, Yulin 537000, China

**Keywords:** thin film transistors, flexible, fully transparent, oxide

## Abstract

Flexible and fully transparent thin film transistors (TFT) were fabricated via room temperature processes. The fabricated TFT on the PEN exhibited excellent performance, including a saturation mobility (μ_sat_) of 7.9 cm^2^/V·s, an I_on_/I_off_ ratio of 4.58 × 10^6^, a subthreshold swing (SS) of 0.248 V/dec, a transparency of 87.8% at 550 nm, as well as relatively good stability under negative bias stress (NBS) and bending stress, which shows great potential in smart, portable flexible display, and wearable device applications.

## 1. Introduction

The field of transparent flexible flat panel displays and wearable sensors with smart, portable, fashion, cool and other advantages have aroused widespread concern in recent years [1,2,3,4,5], as an important component, transparent flexible thin film transistor (TFT) on metal oxides has attracted much attention [3,6,7,8,9,10]. Although the applications of transparent flexible TFTs have broad prospects, some technical problems still restrict the development and application of transparent flexible TFT to a large extent, in which the R & D of the low temperature preparation process of TFT is the most critical. At present, most flexible TFTs are prepared on flexible plastic substrates due to its excellent bendability and reliability, but the flexible plastic substrate is not resistant to high temperature, even for the most heat-resistant flexible polyimide (PI) substrate, the glass transition temperature (Tg) does not exceed 410 °C, also, the translucent nature of yellow leads to its poor application in fully transparent circuits, while the Tg of poly(ethylene 2,6-naphthalenedi-carboxylate) (PEN) and other materials with excellent transparency is only 120 °C [11]. However, the channel layer and electrodes often require thermal annealing process of more than 200 °C to realize the ideal quality and chemical structure of the films, which seriously limits the development of flexible TFT. In addition, most of the current research on flexible TFT focuses on a certain functional layer of TFT, such as electrode, dielectric layer, or semiconductor layer, etc. [12,13,14,15]. However, the process temperature of different functional layers is different, which may easily lead to performance deterioration of some functional layers or the appearance of interface defects, thus reducing the reliability of TFT. Current mainstream solutions are as follows: (i) Developing highly heat-resistant flexible substrate, there are some studies on the transparent flexible PI substrate with high temperature resistance recently [16,17], but the preparation process is difficult and the cost is high, (ii) optimizing the film preparation process to prepare films at low temperatures, especially at room temperature without any thermal annealing process can effectively solve the above problems, it will greatly simplify the process flow and greatly reduce the process cost. However, there are few studies on the preparation of flexible TFT at room temperature now.

In our previous work, we chose metal oxide materials that can be prepared at low temperatures to prepare TFT. Furthermore, high-performance AZO (Al_2_O_3_:ZnO = 2:98 wt%) electrode [13] and indium gallium zinc oxide (IGZO)/ultrathin Al_2_O_3_ channel layer [18] were prepared at room temperature by using optimized Radio Frequency Magnetron Sputtering (RFMS) and Pulsed Laser Deposition (PLD) technologies that enable the deposited atoms to acquire high kinetic energy, but the fully transparent flexible TFT has not been prepared before. In this paper, on the basis of previous research, we have succeeded in fabricating fully transparent, flexible TFTs at room temperature without any thermal annealing process, and then the electrical performance and bending reliability of TFT were characterized and analyzed, the TFT exhibited excellent electrical properties and transparency, relatively good stability under NBS and bending stress, as well as advantages of low cost, and avirulent environmental protection. It brings the industry one step closer to smart, lightweight, cheap, green flexible displays and wearable applications.

## 2. Materials and Methods

**Figure 1:**
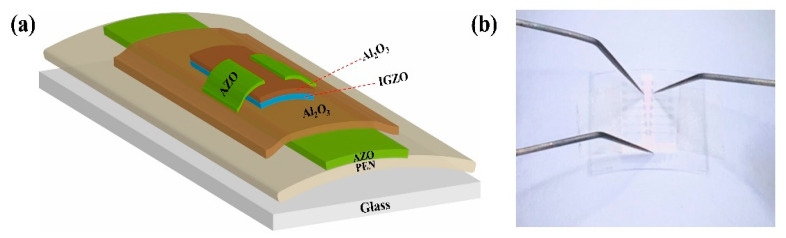
(**a**) Schematic illustration, (**b**) the digital photo.

Figure 1a,b show the schematic and picture of the transparent and flexible TFTs, respectively. First of all, a 90-nm-thick AZO (Al_2_O_3_:ZnO = 2:98 wt%) gate electrode is deposited on PEN by radio frequency (RF) magnetron sputtering with the optimum condition (Power: 80 W, Pressure: 1 mTorr, Atmosphere: pure Ar). Then 320-nm-thick Al_2_O_3_ is fabricated by RF magnetron sputtering, acting as a gate insulator layer. Next, a bi-layer of 8-nm-thick IGZO and 3-nm-thick ultrathin Al_2_O_3_ serve as a channel layer. Finally, a 70-nm-thick AZO (Al_2_O_3_:ZnO = 2:98 wt%) film as S/D electrode is prepared by PLD at the optimized condition (O_2_ flow rate: 0 sccm, pulsing energy: 450 mJ, repeating rate: 5 Hz). The films mentioned above are all patterned by shadow masks and deposited at room temperature, the entire preparation process does not require annealing.

The interface structure, cross-sectional morphology, and composition distribution of TFTs were measured by transmission electron microscopy (TEM, JEOL JEM-2100F). The surface band structure was measured by the X-ray photoelectron emission spectroscopy using Thermo VG ESCALAB 250 photoelectron spectrometer. The electrical characterizations of TFTs were measured by the semiconductor parameter analyzer (Agilent, 4155C). The optical properties of TFTs were measured by an ultraviolet-visible spectrophotometer (shimazu uv-2600).

## 3. Results

**Figure 2:**
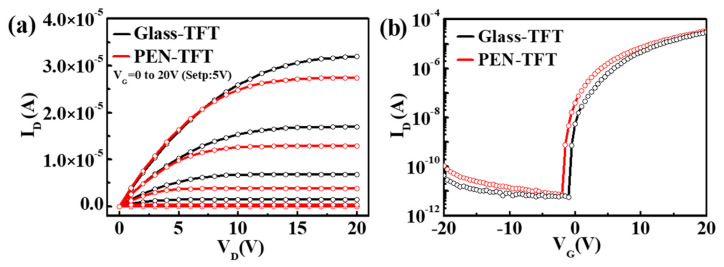
Comparison of (**a**) output and (**b**) transfer characteristics between Glass-TFT and PEN-TFT.

Firstly, we characterize the electrical properties of TFT prepared on a glass substrate and PEN substrate, as shown in Figure 2, there are only slight differences in performance between PEN-TFT and Glass-TFT, which indicates TFTs almost suffer no destruction during transfer and the preparation process to flexible substrates, and the internal stress due to the difference of elasticity between glass and PEN has little effect on TFTs. In addition, this phenomenon indicates that the penetration rate of water/oxygen in the PEN layer is low, which has little impact on the reliability of TFT, there is no need to deposit a buffer layer on the PEN, which is conducive to reducing the technical difficulty and cost. 

**Figure 3:**
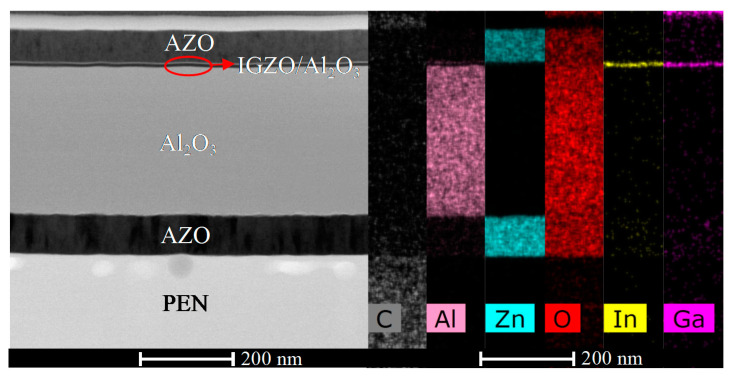
Cross-sectional TEM images and EDS mapping of the TFT.

The PEN-TFT exhibited excellent performance, including a μ_sat_ of 7.9 cm^2^/V·s, an I_on_/I_off_ ratio of 4.58 × 10^6^, a SS of 0.248 V/dec, and a V_th_ of 1.04 V. Figure 3 shows the TEM images of PEN-TFT, which exhibit smooth interfaces between two adjacent layers, the good contact between interfaces can reduce the interface defects and ensure the excellent performance of TFT. Furthermore, the EDS mapping results indicate diffusionless phenomenon and the homogeneous distribution of elemental Al, Zn, In, and Ga, these are the basic conditions for the TFT device to achieve good performance.

**Figure 4:**
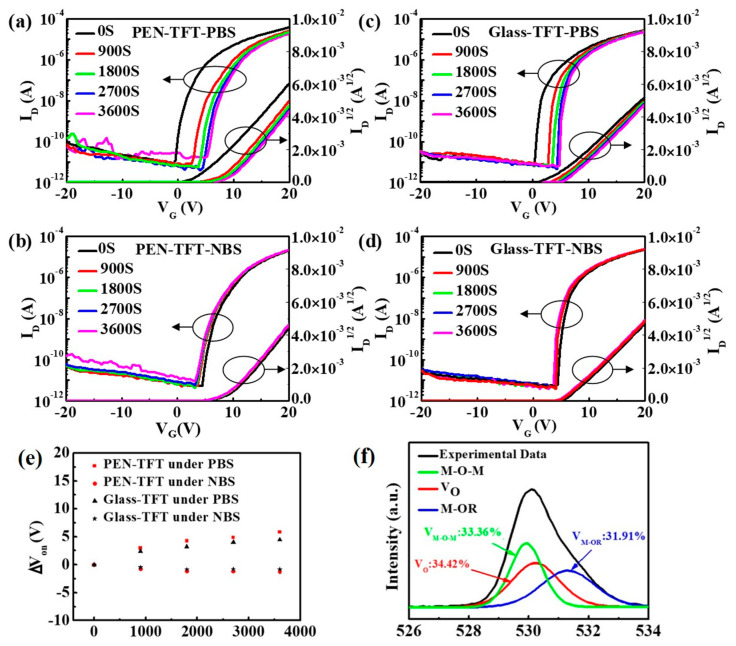
(**a**) PBS and (**b**) NBS results of PEN-TFT, (**c**) PBS and (**d**) NBS results of Glass-TFT, (**e**) the V_on_ shift of these TFTs under PBS (V_G_ = +10 V) and NBS (V_G_ = −10 V), (**f**) O1s photoelectron spectra (XPS) of IGZO/Al_2_O_3_ film.

Figure 4a–d shows the transfer characteristic curves of Glass-TFT and PEN-TFT under positive/negative gate bias stress (positive bias stress (PBS) and NBS) applying V_G_ = +10 V and V_G_ = −10 V for one-hour, respectively. The V_on_ shift under PBS and NBS are compared in Figure 4e. The largest V_on_ shift of the glass-TFT and PEN-TFT under NBS is about −0.9 V, which indicates both Glass-TFT and PEN-TFT possess good NBS stability. However, all TFTs exhibit poor PBS stability with a large positive shift of V_on_ of approximately 5 V. To investigate the reason for the poor PBS stability, the oxygen states of IGZO/Al_2_O_3_ film are measured by X-ray photoelectron spectroscopy (XPS). M-O-M, oxygen vacancies (V_O_) and M-OR peaks centered at ~530, ~531, and ~532 eV are attributed to O^2−^ ions surrounded by metal atoms, the oxide lattice with V_O_, and adsorbed oxygen, respectively [19,20]. As shown in Figure 4f, the high adsorbed oxygen content (31.91%) of IGZO/Al_2_O_3_ film demonstrates channel layer exist numerous adsorbed oxygen, it is well known that adsorbed oxygen can capture electron to form O^−^ or O^2−^, the chemical reaction is shown in Equation (1), and the accumulation of free electrons under PBS will exacerbate the phenomenon, greater positive gate bias is required to enable the TFT to be on. Correspondingly, the chemical reaction of adsorbed oxygen is not promoted under NBS, so the NBS stability is much better than PBS stability [21].

(1) $$O_{2}+e^{-}\to O^{-}+O^{2-}$$

**Figure 5:**
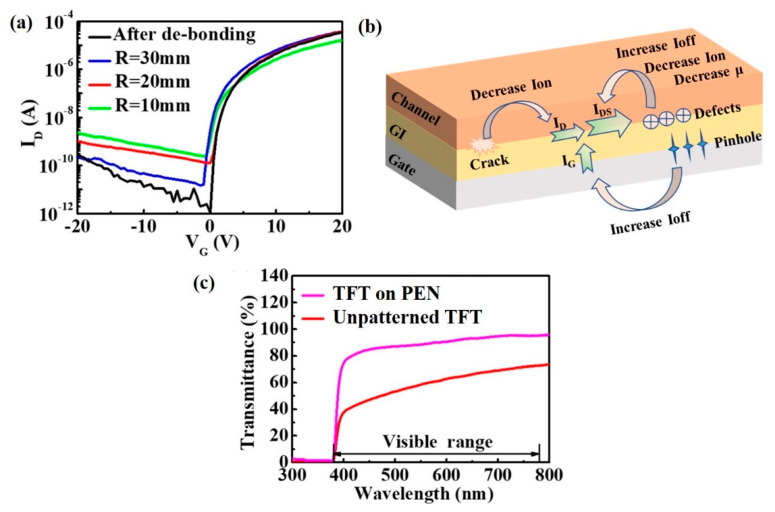
(**a**) Bending test of the flexible VL-TFT, (**b**) schematic diagram of TFT performance degradation under bending, (**c**) transmittance spectra in the wavelength range of 300~800 nm for the flexible TFT on PEN and unpatterned TFT.

The PEN-TFT is measured under various bending radius, and the respective parameters are summarized in Table 1. As shown in Figure 5a, the transfer characteristics show a sharp rise in Ioff as the radius decrease and the mobility of TFT decreases, but the SS increases with the increasing bending radius, Wang et al. reported a similar phenomenon in their research [22]. The degradation of TFT is related to the appearance of interfacial cracks and defects under bending stress. With the increase of bending strain, defects and cracks are likely to occur in stress concentration areas in TFT, such as the interface between dielectric layer (GI) and channel, or the interface between gate electrode and dielectric layer, the mechanism that may cause the degradation of TFT is shown in Figure 5b, the pinhole between gate electrode and dielectric layer generated by bending stress could be a charge transport channel, which will increase the I_G_ and lead the Ioff to increase. Moreover, the defects introduced by bending stress between channel and dielectric layer not only degrade Ion, but also increase Ioff, and it will increase the scattering of carriers, resulting in decreased mobility, as shown in Table 1, the density of interface defects (Nt) increases with the increasing bending stress. In addition, cracks will be generated at the interface between the channel and the dielectric layer under large bending stress, which will weaken the control ability of the gate bias on the conductive channel, resulting in a decrease of Ion.

Figure 5c shows the average transmittance of the PEN-TFT in the visible range can reach 87.8% at 550 nm. The unpatterned TFT (without any mask process) has all the same layers as PEN-TFT and exhibits a transparency of 58.1% at 550 nm. The improvement of transparency may be due to the decreased coverage of the device on the PEN substrate. Transparent PEN-TFT brings the industry closer to transparent, flexible, and green displays.

## 4. Discussion

A comparison of the device in our work with other reported flexible transparent oxide TFTs is summarized in Table 2. According to Table 2, significant advantages of our device can be seen: (i) The fabrication of TFT does not require annealing, which has great advantages in manufacture of flexible circuit, (ii) the TFT has excellent transparency and good electrical properties, which has potential in flexible display and stealth device applications.

## 5. Conclusions

In summary, the flexible transparent TFTs are successfully prepared without any thermal annealing process, the optimized flexible device demonstrates excellent electrical characteristics with a μ_sat_ of 7.9 cm^2^/V·s, an I_on_/I_off_ ratio of 4.58 × 10^6^, a SS of 0.248 V/dec, good stability under NBS, as well as good flexibility. More significantly, the as-prepared flexible device shows excellent transparency (87.8%). This kind of TFT could bring the industry one step closer to smart, lightweight, cheap, green flexible displays and wearable applications.

## Figures and Tables

**Table 1:** Summary of the performance of the flexible TFT after bending.

Radius	Plane	R = 30 mm	R = 20 mm	R = 10 mm
µ_sat_ (cm^2^/V·s)	6	5.6	5.61	4.73
I_on_/I_off_	1.3 × 10^7^	2.63 × 10^6^	3.05 × 10^5^	6.82 × 10^4^
SS (V/dec)	0.237	0.397	0.594	0.723
V_on_ (V)	0.17	−0.88	−0.07	−0.45
I_on_ (A)	5.36 × 10^−5^	4.05 × 10^−5^	4.00 × 10^−5^	1.58 × 10^−5^
I_off_ (A)	4.12 × 10^−12^	1.54 × 10^−11^	1.31 × 10^−10^	2.32 × 10^−10^
Nt (cm^−2^ eV^−1^)	5.01 × 10^11^	9.53 × 10^11^	1.51 × 10^12^	1.87 × 10^12^

**Table 2:** Summary of transparent oxide TFTs prepared on flexible substrates from the literature, including the device presented in this work.

Ref.	This Work	[12]	[23]	[24]	[25]
Substrate	PEN	PEN	PES	PI	PI/SiO_2_
Gate electrodes	AZO (RFMS)	ZnO/AZO (ALD)	ITO	Al (DCMS)	Ti (DCMS)
S/D electrodes	AZO (PLD)	ZnO/AZO (ALD)	IZO (RFMS)	Mo (DCMS)	ITO (RFMS)
Dielectric	Al_2_O_3_ (RFMS)	Al_2_O_3_ (ALD)	Al_2_O_3_ (ALD)	Al_2_O_3_	Al_2_O_3_ (ALD)
Channel layers	IGZO/Al_2_O_3_ (DC/RFMS)	ZnO (ALD)	ZTO (RFMS)	IZO (RFMS)	IWO (RFMS)
Maximum temperature	RT	150 °C	150 °C	300 °C	270 °C
Transmittance of TFT	87.8%	80%	~68%	-	-
µ_sat_ (cm^2^/V·s)	5.61	2	-	6.32	24.86
I_on_/I_o__ff_	3.05 × 10^5^	~10^7^	3.05 × 10^6^	9.7 × 10^7^	~10^5^
SS(V/dec)	0.594	1.4	-	0.39	0.28
Bending radius (mm)	20	Unbending	unbending	20	20
Year	2020	2017	2018	2016	2018

## Data Availability

Data is contained within the article.

## References

[1] Bukke R.N., Mude N.N., Saha J.K., Lee S., Jang J. (2021). P-22: Flexible La Doped ZnO TFTs and Circuits on Polyimide Substrate for Foldable Display. SID Symp. Dig. Tech. Pap..

[2] Cao W., Hsu Y., Jiang Z., Liu F., Wu Y., Zhang X. (2021). High Performance Top Gate Oxide TFT Technology for Large Area Flexible AMOLED Display. SID Int. Symp. Dig. Tech. Pap..

[3] Hu Y., Guo L.Q., Huo C., Dai M., Webster T.J., Ding J. (2020). Transparent Nano Thin-Film Transistors for Medical Sensors, OLED and Display Applications. Int. J. Nanomed..

[4] Ko J.B., Lee S.H., Lee T.I., Lee S., Kim J., Kim H., Kim T.S., Park S.H.K. (2021). Ultrathin, Flexible, and Transparent Oxide Thin-Film Transistors by Delamination and Transfer Methods for Deformable Displays. Adv. Mater. Technol..

[5] Gu Y., Zhang T., Chen H., Wang F., Pu Y., Gao C., Li S. (2019). Mini Review on Flexible and Wearable Electronics for Monitoring Human Health Information. Nanoscale Res. Lett..

[6] Fang G., Li D., Yao B. (2002). Fabrication and vacuum annealing of transparent conductive AZO thin films prepared by DC magnetron sputtering. Vacuum.

[7] Myny K. (2018). The development of flexible integrated circuits based on thin-film transistors. Nat. Electron..

[8] Sharma S., Shriwastava S., Kumar S., Bhatt K., Tripathi C.C. (2018). Alternative transparent conducting electrode materials for flexible optoelectronic devices. Opto-Electron. Rev..

[9] Park H., Oh D.S., Lee K.J., Jung D.Y., Lee S., Yoo S., Choi S. (2020). Flexible and Transparent Thin-Film Transistors Based on Two-Dimensional Materials for Active-Matrix Display. ACS Appl. Mater. Interfaces.

[10] Ha T. (2016). High-Performance Solution-Processed Zinc–Tin-Oxide Thin-Film Transistors Employing Ferroelectric Copolymers Fabricated at Low Temperature for Transparent Flexible Displays. IEEE Electron Device Lett..

[11] Sarma K.R. (2016). Flexible Displays: Substrate and TFT Technology Options and Processing Strategies. Handbook of Visual Display Technology.

[12] Li Y., Yao R., Wang H., Wu X., Wu J., Wu X., Qin W. (2017). Enhanced Performance in Al-Doped ZnO Based Transparent Flexible Transparent Thin-Film Transistors Due to Oxygen Vacancy in ZnO Film with Zn–Al–O Interfaces Fabricated by Atomic Layer Deposition. ACS Appl. Mater. Interfaces.

[13] Zhang H., Li X., Fang Z., Yao R., Zhang X., Deng Y., Lu X., Tao H., Ning H., Peng J. (2018). Highly Conductive and Transparent AZO Films Fabricated by PLD as Source/Drain Electrodes for TFTs. Materials.

[14] Lee J., Park Y.S. (2015). Characteristics of Al-doped ZnO films annealed at various temperatures for InGaZnO-based thin-film transistors. Thin Solid Film..

[15] Hernandez-Como N., Morales-Acevedo A., Aleman M., Mejia I., Quevedo-Lopez M.A. (2016). Al-doped ZnO thin films deposited by confocal sputtering as electrodes in ZnO-based thin-film transistors. Microelectron. Eng..

[16] Ma P., Dai C., Wang H., Li Z., Liu H., Li W., Yang C. (2019). A review on high temperature resistant polyimide films: Heterocyclic structures and nanocomposites. Compos. Commun..

[17] Zhang Y., Qu L., Liu J., Wu X., Zhang Y., Zhang R., Qi H., Zhang X. (2018). Synthesis and characterization of high-temperature-resistant and optically transparent polyimide coatings for potential applications in quartz optical fibers protection. J. Coatings Technol. Res..

[18] Zheng Z., Zeng Y., Yao R., Fang Z., Zhang H., Hu S., Li X., Ning H., Peng J., Xie W. (2017). All-sputtered, flexible, bottom-gate IGZO/Al2O3 bi-layer thin film transistors on PEN fabricated by a fully room temperature process. J. Mater. Chem. C.

[19] Yao J., Xu N., Deng S., Chen J., She J., Shieh H.D., Liu P., Huang Y. (2011). Electrical and Photosensitive Characteristics of a-IGZO TFTs Related to Oxygen Vacancy. IEEE Trans. Electron Devices.

[20] Hosono H. (2006). Ionic amorphous oxide semiconductors: Material design, carrier transport, and device application. J. Non Cryst. Solids.

[21] Jeong J.K., Won Yang H., Jeong J.H., Mo Y., Kim H.D. (2008). Origin of threshold voltage instability in indium-gallium-zinc oxide thin film transistors. Appl. Phys. Lett..

[22] Wang X., Wang M., Jiang W., Zhang D., Wang H., Shan Q. (2018). Mechanical Reliability of Flexible a-InGaZnO TFTs under Dynamic Stretch Stress. IEEE Trans. Electron Devices.

[23] Lee K., Kim Y., Kim J., Oh M.S. (2018). Transparent and Flexible Zinc Tin Oxide Thin Film Transistors and Inverters using Low-pressure Oxygen Annealing Process. J. Korean Phys. Soc..

[24] Zhang L., Huang C., Li G., Zhou L., Wu W., Xu M., Wang L., Ning H., Yao R., Peng J. (2016). A Low-Power High-Stability Flexible Scan Driver Integrated by IZO TFTs. IEEE Trans. Electron Devices.

[25] Tiwari N., Rajput M., John R.A., Kulkarni M.R., Nguyen A.C., Mathews N. (2018). Indium Tungsten Oxide Thin Films for Flexible High-Performance Transistors and Neuromorphic Electronics. ACS Appl. Mater. Interfaces.

